# A consolidated review on stem cell therapy for treatment and management of Alzheimer's disease

**DOI:** 10.1002/agm2.12216

**Published:** 2022-07-25

**Authors:** Faizan Ahmad, Punya Sachdeva

**Affiliations:** ^1^ Department of Medical Elementology and Toxicology Jamia Hamdard University Delhi India; ^2^ Amity Institute of Neuropsychology and Neurosciences Amity University Noida Uttar Pradesh India

**Keywords:** Alzheimer's disease, embryonic stem cells, Induced pluripotent stem cells, mesenchymal stem cells, neural stem cells

## Abstract

Alzheimer's disease (AD) is one of the most common forms of dementia and affects around 50 million people around the globe. AD is diagnosed mainly through imaging techniques and to date only five drugs are approved for management of AD but no promising treatment is available for AD. So in this review, we are focusing on stem cell therapy for AD. This review will cover all stem cells like mesenchymal stem cells, embryonic stem cells, induced pluripotent stem cells, and neural stem cells. Clinical trials of AD have also been discussed. Finally, limitations of stem cells are discussed with ongoing clinical trials, and in the future stem cell therapy can be used for treatment of AD.

## Graphical abstract

Amyloid beta plaques and NFTs are two major hallmark of Alzheimer's Disease. Stem cells like mesenchymal stem cells, embryonic stem cells, pluripotent stem cells and neural stem cells have the potential to combat Alzheimer's Disease and can be the promising therapeutic option in future.

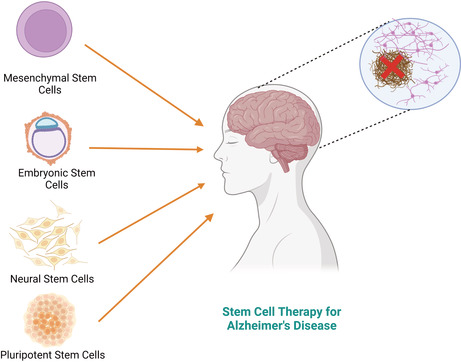

## 1 INTRODUCTION

Memory loss, emotional fluctuations, and functioning impairments are frequent signs of Alzheimer's disease (AD). Dementia, which includes AD, affects 50 million people worldwide, the majority of whom are elderly. AD is the world's fifth biggest cause of mortality, according to the World Health Organization (WHO), with the number of fatalities expected to quadruple by 2050 if present trend continue.^1^, ^2^ Brain imaging, physical and cognitive examinations, laboratory testing, and medical history are used to diagnose AD.^3^ Risk factors of the AD include apolipoprotein E4 (APOE4) genotype and traumatic brain injury, family history and age, obesity, diabetes, hypercholesterolemia, hypertension, and illiteracy. The most prevalent causes of AD are mutations in the genes producing the amyloid precursor protein (APP), presenilin 2 (PSEN2), and presenilin 1 (PSEN1).^4^ As a result, both pharmaceutical and nonpharmacological therapies, such as physical, social, and cognitive activities, should be included in the treatment plan. Lithium and sodium valproate are two medicines used to treat mild to severe cognitive impairment.^5^, ^6^ Nonsteroidal anti‐inflammatory medications (NSAIDs) like naproxen and ibuprofen are also used to treat neuroinflammation and prevent neurodegeneration.^7^, ^8^ Ganstigmine, metrifonate, lecithin, ibuprofen, rofecoxib, latrepiridine, omega‐3 polyunsaturated fatty acids, vitamin B, and vitamin E are among the medications that have failed clinical studies. These drugs have been linked to headaches, nausea, vomiting, neuromuscular dysfunction, and respiratory problems.^5^, ^9^ Only five FDA‐approved medications are now available to treat the symptoms of Alzheimer's disease. Aducanumab was just authorized for use in the year 2021. Memantine (N‐methyl‐D‐aspartate receptor) has been on the market for 10 years (NMDAR antagonist). The remaining five medications are rivastigmine, donepezil (both cholinesterase inhibitors [ChEls]), memantine (an NMDAR antagonist), and galantamine (an NMDAR antagonist).^3^, ^10^ In recent years, stem cell therapy has exhibited significant progress in treating AD, as seen by multiple improvements in clinical studies. Self‐generation, multiplication, division, and reprogramming for multi‐lineage activities are among their many astonishing abilities. Consequently, they can be integrated into existing neural network topologies in this fashion. They also begin to raise the amount of acetylcholine in the brain, which leads to improved memory and cognitive abilities due to the treatment. The primary modes of action for stem cell therapy can be split into two categories based on the ways of action used: endogenous and exogenous mechanisms of action.^11^, ^12^, ^13^, ^14^, ^15^ In most cell‐based treatments, tissue repopulation has been tried through either trans‐difference or the active involvement of injected stem cells, with mixed results. In this review we have discussed different types of stem cells that can be used for treatment of AD.

## 2 GROUPS OF STEM CELLS

**FIGURE 1:**
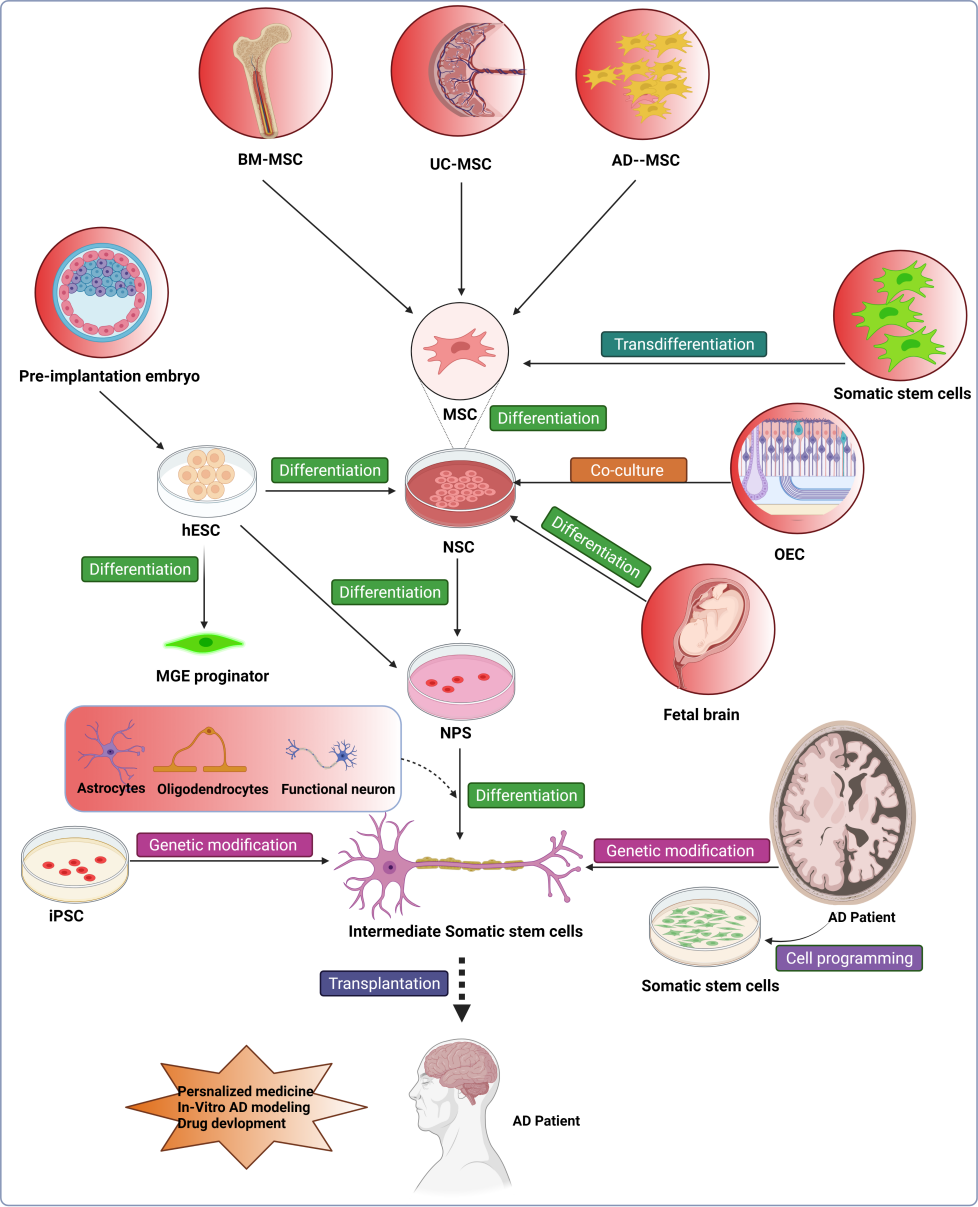
Various stem cells are used to combat Alzheimer's disease‐like Bone marrow‐derived mesenchymal stem cells, Umbilical cord mesenchymal stem cells, Human embryonic stem cells, adipose tissue‐derived mesenchymal stem cells, Induced pluripotent stem cells, Neural stem cells, neural progenitor cells, olfactory ensheathing cells, Medial ganglionic eminence‐like progenitor cells which have the potential to remove amyloid depositions in AD brain.

The selection of the most appropriate cell source is crucial in developing stem cell treatments. Neuronal stem cells (NSCs), embryonic stem cells (ESCs), mesenchymal stem cells (MSCs), and induced pluripotent stem cells (iPSCs) are among the cells now being employed in AD research. Endodermal, mesodermal, and epidermal germs are the three germ layers that give rise to embryonic stem cells. They are called pluripotent cells because they can differentiate into a wide range of cell types when given the opportunity. In embryonic days 5 and 6, pluripotent stem cells (PSCs) are formed from the growing blastocyst's core cell mass. They are dubbed pluripotent stem cells because they specialize in any cell type, including the immune system.^12^, ^15^, ^16^ MSCs, derived from Wharton's jelly of umbilical cord blood, are involved in the formation of mesenchymal tissue and can be obtained from these sources (UCB‐MSCs). These adult stem cells can also be found in other adult stems cell habitats, such as adipose tissue and bone marrow. NSCs, on the other hand, are multipotent cells that can differentiate into any neural cell during the development of the embryo. Finally, iPSCs are derived from mature somatic cells, primarily adult dermal fibroblasts, which have had their DNA changed to make them mirror ESCs and PSCs in terms of phenotypic and differentiation potential through the use of viral vectors or minor chemical treatments, respectively.^17^, ^18^ Because stem cells promote the renewal and replacement of tissues and cells, they have therapeutic effects. Currently, there are two approaches that can be used to treat patients with stem cell. The first is to stimulate the production of indigenous stem cells, and the second is to transplant stem cells into damaged cells or tissues to restore them. AMD3100, allopregnanolone (APAP), granulocyte, fluoxetine, stromal cell‐derived factor‐1a (SDF‐1a), and colony‐stimulating factor (G‐CSF) are examples of chemical substances and stem cell stimulating factors that can stimulate the production of endogenous stem cells and have neuroprotective effects.^19^ The increased activity of endogenous neural precursor cells (NPCs) and promotion of the presence of newly generated cells resulted in significantly more BrdU+ cells in 3xTgAD mice and improved cognition and monomorphism. Another study used three different chemicals to stimulate the production of hematopoietic progenitor cells already present in the patient's body (HPC). As well as SDF1af, GCSF, and AMD3100, an antagonist of the CXCR4 receptor assisted in mobilizing and migrating bone marrow–derived hematopoietic progenitor cells (BM‐HPCs) into the brain.^20^ Treatment with fluoxetine has demonstrated that NSCs can develop into neurons and protect them against amyloid‐mediated cell death. It has recently been demonstrated that transplanting stem cells from the human umbilical cord, amniotic membrane‐derived epithelial cells, and mesenchymal stem cells into the brains of transgenic Alzheimer's rats can help reduce the symptoms of the disease. As a result, the amyloid, APP production, and microglia activation levels were all lowered. Cognitive and memory capacities improved due to the treatment, and neuron lifespan was extended. Another study claimed that injecting stromal cell–derived factor 1 into Alzheimer's transgenic mice had a therapeutic effect on lowering the amount and pace of amyloid production by peripheral mononuclear cells, which were then converted into microglia.^21^ Patients with AD have demonstrated that stem cell transplantation can help them regain their function. MSCs can aid in the survival of AD cell models, increase metabolic activity, and aid in their release. The co‐culture of BV‐2 and human MSCs and the exposure of mouse microglia to amyloid resulted in increased synthesis of neprilysin, an enzyme that degrades amyloid. It has been demonstrated that MSC‐derived stem cell transplantation from human and mouse MSCs can reduce amyloid deposition, improve cognition, and slow the progression of Ad in mouse models. Mouse neural stem cells were colonized around amyloid plaques and genetically modified to activate metalloproteinase 9 (MMP9), a secreted protease demonstrated to degrade aggregated Ab peptides in the laboratory. However, after being implanted into the AD mouse brain, these NSCs have not spread to other parts of the brain. In addition, because pro‐inflammatory components are reduced, ADSCs have been shown to alleviate AD pathogenesis by decreasing amyloid breakdown and memory progress. Human amniotic epithelial cells showed no signs of immunological rejection and survived for 8 weeks after being transplanted (HSECs). HAEC transplantation improved contiguous memory deficits in transgenic mice while simultaneously boosting acetylcholine levels and cholinergic neuritis in the hippocampus. More investigation is also required to establish the optimal conditions for maximizing AD pathology.^14^ A variety of stem cells for AD are depicted in Figure 1.

## 3 ESCs FOR AD

When ESCs are formed by the internal cell mass of pluripotent blastocysts, they are characterized as pluripotent because they can differentiate into ectoderm, mesoderm, and endoderm cell forms, among other cell types. According to the study's findings, ESCs effectively improved spatial learning and memory in AD rats by converting gamma‐aminobutyric acid neurons and cholinergic neurons derived from basal forebrain neurons, which were found to be effective in improving spatial learning and memory. Because of their poor teratoma establishment, unpredictable immune response, and rejection, ESCs have limited clinical usefulness. Furthermore, before they can be used in clinical research approved by the Food and Drug Administration, it is necessary to overcome the ethical disputes due to the development of these technologies.^22^ Experiments with experimental ESCs have been conducted in various mouse models with varying degrees of success. The ability of embryonic stem cells to develop into any cell type is the most significant advantage they possess. The potential of ESCs to differentiate in various orientations and the ability of ESCs to form tumors are both significant drawbacks of using them in research and clinical practice. Cholinergic basal forebrain neurons (BFCNs), severely damaged in AD patients, have been generated from human embryonic stem cells (hESCs), frequently used in this process. Additionally, mouse embryonic stem cells (mESCs) have gained widespread acceptance. In the Alzheimer's rat model, the transplantation of these neurons results in the creation of ESCs and the development of neural precursor cells, which can later be employed to treat the animals. The rats demonstrated significant improvements in their memory impairments compared to controls regarding behavioral changes. According to current research findings, hESCs can develop into cholinergic neurons that connect to existing neuronal networks in the vitreous and hippocampus tissues. An experiment similar to this was conducted using mESCs and hESCs injected into mature BFCNs. After the cells were transplanted into AD mice, researchers discovered that the animals' memory and learning efficiency had been altered. However, there has been progress in the differentiation of hESCs into progenitor cells of the medial ganglion protrusion, which is essential because the medial ganglion protrusion (MGE) is critical in the development of the embryonic brain because it is responsible for the initialization of basal forebrain neurons (including BFCNs and intermediate gamma‐aminobutyric acid neurons). In this study, the reintroduction of these MGE‐like progenitor cells into the hippocampus of mice was highly comparable to those of the previous study, which indicated that the cells were capable of producing MGE.^14^, ^21^, ^22^

## 4 NSCs FOR AD

NSCs inside the adult brain are found in the subgranular zone (SGZ) and subventricular zone (SVZ).^13^ NSCs can differentiate into cells, including neurons, oligodendrocytes, and astrocytes. NSCs may additionally be contingent or distinguished from ESCs and iPSCs, from postmortem and fetal neonatal brain tissue. In animal AD models, transplanted NSCs are distinguished into developed brain cell forms.^23^ The grafted cells should be dispersed across artificial tissue for adequate neuronal replacement and then introduced into the functional environment of the host brain. However, it is unclear whether NSCs can evolve into unique types of neural cells. The recipient ecosystem was greatly affected by the differentiation and migration of grafted NSCs. However, there is frequent unwanted differentiation with NSCs recorded in nonneuronal glial cell types.^24^ To what extent neuronal replacing leads to the positive results of transplant with NSC is not clear. The paracrine result obtained when NSC transplantation is over has secured encouragement over cell replacement, as in other stem cells. To rescue cognitive function in AD, attained from brain neurotrophic factor (BDNF) emitted from NSCs is salient. Moreover, it has been stated that NSC transplantation has neuroregenerative, neuroprotective, and immune‐modulatory roles. Further investigations using NSC transplantation warrant tumorigenesis and functional recuperation.^25^ There have been reports of induced NSCs originating from sertoli cells, astrocytes, and fibroblasts. NSCs based on ESCs have been studied and differentiated into similar astrocytes. However, the iNSCs succeeding transplantation in vivo possibility is yet known to be inconstant. Thus, NSCs may be utilized as a revamped technique as distribution mediums to bear curative agents like neprilysin. NSC‐based drug delivery therapy, a neuronal replacement, has recently attained attention.^24^, ^25^

## 5 MCSs FOR AD

In their capacity as pluripotent cells, MSCs can differentiate into a diverse spectrum of cell types, which is why they are categorized as bone marrow MSCs (BMSCs), umbilical cord MSCs (UC‐MSCs), adipose‐derived MSCs (AD‐MSCs), and amniotic fluid MSCs (AF‐MSCS).The Wharton jelly from umbilical cord blood is used in the laboratory to grow the different forms of mesenchymal tissue in various ways. Adult stem cells, such as fat cells and neural stem cells, are found in a range of tissues and organs and adipose (fat) tissue and bone marrow. It is generally agreed that the germ layer of the mesoderm is a representation of the beginning of the embryonic development process. Differentiation capacity and appearance of bone marrow MSCs can vary depending on the type of donor tissue used in the transplantation procedure.^26^ Umbilical cord blood is retained after a baby is born, and it is used to diagnose and treat newborns, the blood taken from the placenta after the baby is delivered. Umbilical cord blood is used to treat a variety of conditions. MSCs also contain a high quantity of hemoglobin, which is beneficial. When employing mouse AD models, a prior study on UC‐MSCs revealed that UC‐MSCs could increase regular exploration and help to prevent a decline in cognitive function. Another effective route for MSCs to engage in tissue regeneration is releasing extracellular vesicles and microvesicles, which are most typically seen in the environment and are the most commonly discovered in the body. It is possible to change the composition of BMSCs by genetic modification.^27^, ^28^, ^29^ MSC‐derived extracellular vesicles loaded with therapeutic medicines, such as siRNAs and enzymes, can be used to eliminate amyloid from the body. According to the findings, MSC‐derived EVs can dramatically halt the progression of AD in 5XFAD mice, who showed considerable improvement in cognitive tests compared to 5XFAD mice treated with saline. Cell magazine presented the outcomes of the study. Control mice had much higher amyloid plaque loads in their hippocampus, whereas mice treated with EV had significantly reduced amyloid plaque loads.^30^ Researchers can train MSCs to express high pro‐inflammatory cytokines and vascular endothelial growth factors. The cells can then restore function in an AD model if the researchers wish to accomplish it. MSCs are the most widely used stem cells. Despite ethical concerns, they are being used in various AD research studies, particularly productive cord blood banks, because MSCs are relatively easy to select and manage if fetched later than standard delivery compared to other types of stem cells. MSCs are also relatively inexpensive compared to other stem cells, making them an attractive option for research. Despite ethical issues, they are being used in many AD research projects, particularly productive cord blood banks, because MSCs have the most significant potential for regeneration.^31^, ^32^

## 6 iPSCs FOR AD

A total of four different transcription factors, namely OCT4, SOX2, c‐MYC, and KLF4, are active in creating induced pluripotent stem cells. Each transcription factor is responsible for a specific function (iPSCs). They can be made by heterologous human cells, which can develop into any cell, such as glial cells or neurons, and are therefore useful in research. IPSCs, in particular, have the potential to hold significant promise in a variety of applications, including the treatment of AD and the modeling of disorders. This is due to their ability to overcome the limitations associated with immunological rejection and the ethical difficulties of using other stem cell types in humans.^33^, ^34^, ^35^ In the initial development of the induced pluripotent stem cell (iPSC) AD model in fibroblasts from Alzheimer's patients with PS1 (A246E) mutations and PS2 (N141I) mutations, the ability to differentiate between neurons and those with the PS1 mutations and PS2 mutations is a distinguishing feature. Using neurons generated from Alzheimer's patients and iPSCs, the researchers discovered that they could boost the amyloid‐42 produced by mutant PS1/PS2 animals. Several specific inhibitors and modulators of the enzyme have been discovered, raising the possibility that they could be utilized to target neuronal amyloid 42 synthesis in developing AD therapeutic drugs in the future. It was decided to take advantage of the most current advancements in stem cell technology to generate iPSCs from AD patients who had the APP gene duplicated in their brains. Also discovered was that the neurons recovered from these iPSCs had significantly higher balanced levels of amyloid phospho‐tau and active glycogen synthase kinase‐3 than those recovered from all other iPSCs, indicating that they had developed a more mature nervous system than the neurons recovered from all other iPSCs.^12^, ^36^, ^37^, ^38^ In iPSC‐derived neurons, a significant decrease in phospho‐tau and active glycogen synthase kinase‐3 was observed following treatment with the drug; a significant decrease in phospho‐tau and active glycogen synthase levels was observed in kinase‐3 in iPSC‐derived neurons was also observed following treatment with the drug like donepezil, galantamine, and rivastigmine.^39^ Exogenous genes and retroviral and lentiviral vectors are incorporated into the genome of induced pluripotent stem cells (iPSCs), to express reprogramming factors such as the OCT4, SOX2, c‐MYC, and KLF4, to investigate the possibility of results in genetic disorder and cancer. Following the study's findings, it is conceivable to employ induced pluripotent stem cells to investigate disease characteristics specific to AD, with the results indicating that this is a viable option. Consequently, iPSCs generated from retroviral or lentiviral vectors cannot be employed in transplantation therapy due to their incapacity to differentiate. Researchers have manufactured isogenic human pluripotent stem cell lines at breakneck speed and with pinpoint accuracy thanks to the development of CRISPR/Cas9 (Clustered Regularly Interspaced Short Palindromic Repeats/Cas9) technology. In order to achieve the desired results, CRISPR‐Cas9 is most typically used on neural organoid founder cells such as ESCs and iPSCs to achieve the desired results.^40^ With the development of CRISPR/Cas9 genome editing technology, it has become possible to create isogenic controls, which can be achieved by correcting single AD‐causing repairable mutations. Potentially feasible approaches include self‐organizing 3D cerebral organoids made of iPSC‐derived cells, which exhibit significant aspects of brain‐specific cytoarchitecture and network dynamics and the investigation of complex neural network phenomena in these AD models, among other things. The in vitro models of AD have revealed a plethora of pathophysiological components of the disease, which can be used to evaluate future treatment possibilities for the condition. Using non‐integrating episomal vectors, researchers were able to create virus‐free human induced pluripotent stem cells (iPSCs) from AD patients with the PS1 mutation (A246E).^41^, ^42^, ^43^, ^44^ A246E mutations in the PS1 gene of AD patients and A246E get deposits in the AD patient brains. According to the National Institute on Aging, humanized AD models can be developed utilizing induced pluripotent stem cells with the PS1 gene mutation. Scientists hope that these models will aid in understanding the pathogenesis of AD and the screening of prospective treatment medications. iPSC‐derived neurons cannot be directly implanted into AD patients at this time due to defects in the genes encoding proteins required for proper neuron function. These defects are present in the genes encoding the APP, PS1, and PS2 proteins required for proper neuron function. It has recently been established that a range of genetic techniques, such as homologous reconnection, may be used to fix mutations in mutant iPSCs, and that this challenge can be overcome in this way. AD patients may one day be able to receive transplants of neuronal cells that have been genetically modified to fix genetic mutations.^35^, ^45^, ^46^ As reported by the researchers, a mutation in PSEN1 caused an increase in amyloid‐beta production and oxidative stress during the formation of astrocytes and changes in cytokine release and Ca^2+^ homeostasis during the cell's growth. The ability of PSEN1 astrocytes to give brain support during astrocyte formation was compromised as a result. Research by Kondo et al. and Israel et al. found that the accumulation of amyloid‐beta oligomers in the brain due to individual iPSC cell injury is associated with greater reactive oxygen species (ROS).^41^ According to the researchers, in this way, they may be able to develop a beneficial treatment model and testing ground for determining the optimal pharmacological regimen for AD patients while also analyzing the biology of the disease, which would be beneficial for both patients and researchers.^38^ Clinical trials of stem cell therapy for AD management are mentioned in Table 1.

**TABLE 1:** Clinical trials of stem cell therapy for AD management

Trial number	Cell source	Sponsor	Study phase	The route of administration	Criteria for Eligibility of Trial	Primary Outcome	Secondary Outcome	Date of starting trial	Date of ending trial	Location	Time frame	Reference number
NCT03172117	h UBC‐MSC	Medipost Co.Ltd.	Phase 1&2 (randomized quadruple blind controlled)	Intraventricular	Diagnosis of probable Alzheimer type according to NINCDS‐ADRDA criteria and K‐MMSE score of 18–26 at visit 1	Change from the baseline in ADAS‐Cog	Change from the baseline in S‐IADI, k‐MMSE, CGA‐NPI, and so forth	2017–05	2021–12	Korea	24 months	^47^
NCT02672306	h UBC‐MSC	South China Research Centre for stem cell and Regenerative Medicine	Phase 1&2	Intravenous	A diagnosis of probable AD and mixed dementia according to the NINCDS‐ADRDA,MMSE score between 3 and 20, both inclusive	Change in ADAS‐Cog score	Change in ADCS‐CCGIC score, MMSE,ADCS‐ADI, and so on	2016–05	2019–10 (Not yet recruiting)	China	10 weeks	^47^
NCT02600130	Longeveron MSC	Longeveron LLC	Phase 1	Peripheral intravenous	At the time of enrollment, be diagnosed with AD in accordance with the NINCDS‐ADRDA criteria: MMSE score between 18 and019	Incidence of any serious adverse events	Neurologic/neurocognitive assessments,ADAS‐Cog 11,abd so forth	2016–08	2019‐10 (recruiting)	USA	2,4,13,26,39,52 weeks	^47^
NCT02054208	Hucb‐msc	Medipost Co.Ltd	Phase 1 or2 (randomized quadruple blind controlled)	Intraventicular	Diagnosis of probable Alzheimer type according to NINCDS‐ADRDA criteria and K‐MMSE score of 18–26 at visit 1	Numberif subjects with adverse events	Change from the baseline in ADAS‐Cog, S‐IADL, K‐MMSE, CGA‐NPI, and so forth	2014–02	2019–07	Korea	42 months	^47^
NCT01696591	Hucb‐MSC	Duk Lyul Na	Phase 1 (case controlled)	Brain surgery	Subject who have enrolled in NCT01297218 and who have similar characteristics	Incidence rate of adverse events	ADAS‐Cog response rate	2012–03	2013‐09 (unknown)	Korea	24 months	^47^
NCT01617577	Filgrastim (G‐CSF)	University of South Florida	Phase 1&2 (crossover)	Subcutaneous	People with probable AD (by NINCDS‐ADRDA criteria);MMSE score between 10 and 24	Cognitive measures including ADAS‐Cog, selected CANTABS tests	None	2009–06	2012‐02 (compleeted)	USA	2,4,14 weeks	^47^
NCT0154768689	hUCB‐MSC	Affiliated Hospital to the Academy of military Medical Sciences	Phase 1&2 (open)	Intravenous	Probable Alzheimer's disease as determined by NINCDS‐ADRDA criteria;MMSE score between 3 and 20, both inclusive	Numberof participant with adverse events	Change from the baseline in ADAS‐Cog at 12 weeks postdose	2012–03	2016–12 (active, not recruiting)	China	10 weeks	^47^
NCT01297218	hUCB‐MSC	Medipost Co.Ltd.	Phase 1	Intravenous	Dementia as determined by DSM4 criteria; probable Alzheimer's disease as determined by NINCDS‐ADRDA criteria; K‐MMSE score in the range of 10 to 24	Number of participant with adverse events	Change from the baseline in ADAS‐Cog at 12 weeks postdose	2011–02	2012–12 (completed)	Korea	12 weeks	^47^
NCT03117738	NA	NA	NA	NA	NA	ADAS‐Cog	MMSE, CDR‐SB, NPI, GDS, ADL, biomarkers (MRI, Amyloid Beta)	2017–19	Active,NR	United State	32 weeks	^47^
NCT02600130	NA	NA	NA	NA	NA	AE number	ADAS‐Cog, ADL, Biomarkers (CSF,Amyloid Beta)	2019–21	Active,NR	United State	65 weeks	^47^
NCT04040348	NA	NA	NA	NA	NA	AE number	ADAS‐Cog, MMSE, NPI, Diverse biomarkers	2019–21	Recruiting	United State	65 weeks	^47^
NCT03724136	NA	NA	NA	NA	NA	MMSE, ASQ‐SE	Activities of daily living	2018–22	Recruiting	United State	12 months	^47^
NCT02672306	NA	NA	NA	NA	NA	ADAS‐Cog	ADAS‐Cog, CIBIC, CIBIC plus, MMSE, ADL, NPI biomarkers (plasma Amyloid Beta)	2017–19	Active,NR	China	36 weeks	^47^
NCT01547689	NA	NA	NA	NA	NA	AE number	ADAS‐Cog, MMSE,CIBIC, ADL, NPL, biomarkers (Amyloid Beta)	2012–16	Unknown status	China	10 weeks	^47^

## 7 CONCLUSION AND FUTURE DIRECTIONS

Even while research has made considerable strides in recent years, a medication that can prevent or delay the onset of dementia or perhaps even slow its progression is currently unavailable. Because various distinct pathways can trigger AD, multiple therapy options for the condition are becoming increasingly diverse. Using stem cells as a treatment option has been increasingly popular due to their capacity to target specific pathways and improve patient outcomes. Many people are turning to stem cell therapy for AD, which is getting increasingly sophisticated in its treatment even if it is still in its early phases. Many elements should be considered while making plans for the future, including the design and execution of any future clinical research that may be conducted. Identifying the most effective stem cell and transplantation technique for achieving massive transformation in the second phase is critical for achieving significant transformation in the first place, as is determining the most effective stem cell and transplantation technique for achieving massive transformation in the first phase. Several stem cells have been discovered that have exhibited potential benefits in animal studies. The maximum ability for differentiation combined with moderation in the segregation process of MSCs has been established as the standard for stem cell clinical trials in humans. As a result, because the efficacy of MSCs has not yet been established, consideration must be given to the kind of stem cell used in research and the source of the stem cell during the study process. When addressing stem cells in clinical practice, several critical concerns must be taken into account. Among these are cell differentiation, cell proliferation and integration, cell migration (including oneness), neural stem cells, and the introduction of stem cells into human bodies, among other things. According to the preceding paragraph, there is sufficient reason to be concerned about the potential negative consequences of this decision's execution after it is implemented. Even though there were no detrimental effects seen throughout the 24 months following cerebral human MSC migration, it is recommended that additional study into the efficacy and protection given by stem cell therapy be done shortly. When patients suffering from ischemic stroke were given intravenous MSC transplantation as part of a clinical trial, it was discovered that the use of MSCs to treat a variety of human diseases had no significant immediate or long‐term nonbeneficial consequences, no long‐term adverse events. Also noteworthy is that, by optimizing the design of clinical investigations, it is possible to increase the number of clinical outcomes attained in a given period in a controlled environment. Increased funding will increase the number of stem cell trials available to patients suffering from a less severe stage of AD or those suffering from the disease in its early stages. Patients suffering from mild to moderate AD will be recruited more significantly as funding becomes available. According to current expectations, incorporating newly developed in vivo biomarkers into clinical research would provide greater power and insight into patient selection and quantity efficacy in clinical research than previously possible using previously recognized marker types. Cell extraction and injection improvements can be approached from various perspectives when working with the immune system, neuro factors, enzymes, proteins, and gene therapy, to name a few examples of applications, among many others. Additionally, it is vital to analyze ethical considerations at every stage of creating a stem cell–based technology at every level of the process as part of the overall development procedure for the technology.

## AUTHOR CONTRIBUTIONS

FA, manuscript writing and drawing figures and PS, manuscript reviewing and editing.

## FUNDING INFORMATION

Not received.

## CONFLICT OF INTEREST

The authors declare they have no conflict of interest.

## CONSENT FOR PUBLICATION

All authors have given consent for publication.
